# Main indications, diagnostic and therapeutic yield of bronchoscopy in the ICU

**DOI:** 10.1186/cc12089

**Published:** 2013-03-19

**Authors:** J Iglesias, B Gonzalez, O Rajas

**Affiliations:** 1 La Princesa Hospital, Madrid, Spain

## Introduction

Bronchoscopy (BC) is a frequently used diagnostic and therapeutic tool in our ICU. We planned to review the patients and conditions in which the procedure was performed. The objective was to determine indications, diagnostic and therapeutic yield.

## Methods

A retrospective, single-center observational study was carried out. All BC performed in a 20-bed polyvalent ICU of a tertiary university hospital over a period of 30 months (from January 2010 to June 2012) were included. The main variables collected were demographic data and clinical data including comorbidities, endobronchial techniques and results.

## Results

A total of 470 BC were carried out in 240 patients admitted to the ICU (68% were male and 32% were female). All of the procedures were performed in mechanically ventilated patients. The mean patient age was 61 ± 14 years, with an APACHE II score at admission of 20 ± 7. The main cause for ICU admission was respiratory failure (27%). The most common comorbidities were smoking, chronic obstructive pulmonary disease, cardiovascular risk factors and atrial fibrillation. The main indications for performing the BC were percutaneous tracheostomy endoscopically guided (28%), resolution of atelectasis (27%), aspiration of secretions (24%) and hemoptysis (10%). Bronchial brushing was performed in 435 cases (93%) obtaining microbiological growth in 64% of them, mainly *Pseudomonas aeruginosa *and *Acinetobacter baumanii*. Bronchoalveolar lavage was performed in 62 cases (13%) obtaining microbiological growth in 61% of them, mainly cytomegalovirus and *Stenotrophomona maltophilia*. Citology was performed in 60 cases (13%); 7% showed the presence of microorganism and 13% revealed precancerous/cancerous tissue changes.

## Conclusion

BC can be performed at the bedside, avoiding transfers outside the ICU that may cause clinical worsening. Most frequent indications were bronchoscopy-guided percutaneous tracheostomy, resolution of atelectasis, aspiration of secretions and hemoptysis. Microbiological results obtained revealed the presence of aggressive pathogens. BC in ICU provides valuable diagnostic information and has therapeutic utility.
